# Cellular functions of TMEM16/anoctamin

**DOI:** 10.1007/s00424-016-1790-0

**Published:** 2016-01-25

**Authors:** Uhtaek Oh, Jooyoung Jung

**Affiliations:** Sensory Research Center, CRI, College of Pharmacy, Seoul National University, Seoul, 151-742 South Korea; Department of Molecular Medicine and Biopharmaceutical Sciences, Graduate School of Convergence Science and Technology, Seoul National University, Seoul, 151-742 South Korea

**Keywords:** Anoctamin, TMEM16, Ca^2+^-activated Cl^−^ channel, Scramblase, Cl^−^ secretion, Nociception, Tumorigenesis, Proliferation

## Abstract

Ca^2+^-activated Cl^−^ channels (CaCCs) are a class of Cl^−^ channels activated by intracellular Ca^2+^ that are known to mediate numerous physiological functions. In 2008, the molecular identity of CaCCs was found to be anoctamin 1 (ANO1/TMEM16A). Its roles have been studied in electrophysiological, histological, and genetic aspects. ANO1 is known to mediate Cl^−^ secretion in secretory epithelia such as airways, salivary glands, intestines, renal tubules, and sweat glands. ANO1 is a heat sensor activated by noxious heat in somatosensory neurons and mediates acute pain sensation as well as chronic pain. ANO1 is also observed in vascular as well as airway smooth muscles, controlling vascular tone as well as airway hypersensitivity. ANO1 is upregulated in numerous types of cancers and thus thought to be involved in tumorigenesis. ANO1 is also found in proliferating cells. In addition to ANO1, involvement of its paralogs in pathophysiological conditions was also reported. ANO2 is involved in olfaction, whereas ANO6 works as a scramblase whose mutation causes a rare bleeding disorder, the Scott syndrome. ANO5 is associated with muscle and bone diseases. Recently, an X-ray crystal structure of a fungal TMEM16 was reported, which explains a precise molecular gating mechanism as well as ion conduction or phospholipid transport across the plasma membrane.

## Introduction

Cl^−^ channels activated by Ca^2+^ are collectively called Ca^2+^-activated Cl^−^ channels (CaCCs). CaCCs are found in a variety of species ranging from invertebrates to mammals. In addition, activity of CaCCs was observed in almost all tissues. The wide distribution of CaCCs in various tissues indicates its diversity in physiological functions. However, a detailed description of their functional roles was not obtained before a molecular identity of CaCCs was discovered. The biophysical properties of CaCCs were well described in a *Xenopus* oocyte, where CaCCs are important for blocking polyspermy [57]. One of the best known functions of CaCCs in mammals is the Cl^−^ secretion in secretory epithelia [39, 56]. In line with this, activities and properties of CaCCs were described in many secretory epithelia such as airway, salivary gland, pancreatic ductal cells, and intestines [39, 56]. CaCC action is not limited to Cl^−^ secretion in epithelia. CaCC activity was found in many excitable tissues such as smooth muscles, cardiac muscles, olfactory sensory neurons, and somatosensory neurons, too [6, 27, 53, 54, 60, 75, 76, 120]. CaCCs are activated by intracellular Ca^2+^ exhibiting an outwardly rectifying current-voltage relationship at relatively low Ca^2+^ [57, 58]. Ca^2+^-activated currents are voltage dependent and show a greater current amplitude in a depolarization state than at hyperpolarization.

A candidate gene, TMEM16A, for CaCCs was cloned by three groups with different cloning strategies [18, 94, 116]. TMEM16A was renamed as anoctamin 1 (ANO1) because it was predicted to have eight transmembrane (TM) domains [116]. ANO1 has nine additional paralogs ranging from ANO2 (TMEM16B) to ANO10 (TMEM16K). ANO1 follows the biophysical and pharmacological properties of CaCCs. ANO1 is blocked by nonselective Cl^−^ channel blockers as well as relatively specific blockers to CaCCs [116]. ANO1 and ANO2 are activated by intracellular Ca^2+^ at the physiological range [45, 86]. Whether other paralogs are activated by physiological concentration of Ca^2+^ is not clear [32]. Thus, only ANO1 and ANO2 are considered as CaCCs.

Among those 10 proteins, ANO1 has been most extensively studied so far. It is involved in many physiological functions such as fluid secretion in many secretory epithelia, smooth muscle contraction, nociception, and most surprisingly, tumorigenesis and cell proliferation. ANO2 has been found in olfactory bulb; thus, a role in mediating olfaction was suggested [87, 99]. However, a genetic ablation of ANO2 in olfactory sensory neurons fails to show a phenotype for olfaction [11]. ANO2 is also expressed in the hippocampus and modulates a synaptic transmission in the brain [45]. ANO3 is expressed highly in dorsal root ganglion (DRG) neurons controlling nociception. ANO5 is mainly found in muscles and bones [72]. A missense mutation of *Ano5* is associated with gnathodiaphyseal dysplasia, an autosomal dominant inherited bone disorder [108], and muscular dystrophy or myopathy [12]. However, ANO5 is not expressed in the plasma membrane and is not active as a channel [108]. ANO6 is a scramblase that transports phospholipids bidirectionally between the two leaflets [102, 103, 117, 119]. The scramblase activity of ANO6 is Ca^2+^ dependent. ANO4, ANO8, and ANO9 also show scramblase activity [102]. A mutation of *Ano6* that truncates the ANO6 protein is associated with a rare bleeding disorder, the Scott syndrome [103]. Functions of other anoctamin family genes have not been well described until now. These functions of ANO family genes are discussed in details with focus on tumorigenesis and cell proliferation.

## Mechanisms of activation

**Fig. 1:**
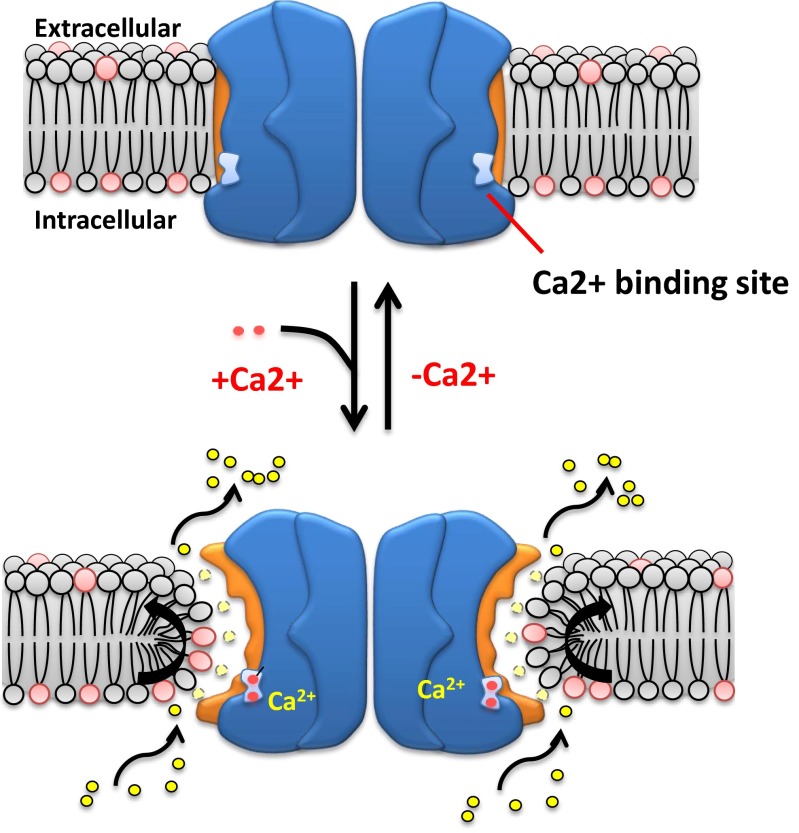
A schematic diagram illustrating the scramblase and ion-conducting mechanism of anoctamin family. When the subunit cavity opens after Ca^2+^ binding, phospholipids and ions are transported through the subunit cavity

Since ANO1 was cloned, the mode of Ca^2+^ action or the Ca^2+^ binding site of ANO1 was proposed by many scientists [106, 114, 118]. A mutagenesis study revealed that mutations of Glu residues markedly shifted the dose-response curve of Ca^2+^ in activating ANO1 [106, 118]. These Glu residues were later found to consist of the Ca^2+^ caging residues [14]. Recently, the X-ray crystal structure was discovered [14]. Brunner and colleagues found a TMEM16 gene in fungus, *Nectria haematococca* (nhTMEM16), which functions as a phospholipid scramblase activated by Ca^2+^, but not as a channel. After crystallization, the protein structure of nhTMEM16 at ∼3.5 Å resolution was obtained. The functional nhTMEM16 is a dimer consisting of two identical subunits. Each subunit has 10 TM helices instead of 8. When ANO1 was cloned, anoctamin 1 was named after its eight-TM domain topology because all programs in the public domain predicted an eight-TM domain topology for TMEM16A [116]. In the lateral side of each subunit, there is a narrow crevice that spans the entire membrane. This cavity is called the *subunit cavity* [14]. The surface of the subunit cavity is hydrophilic even though it is buried in the plasma membrane. The Ca^2+^ binding site is located in the subunit cavity at a distance of one third of the membrane from the intracellular surface. In the subunit cavity of nhTMEM16, five acidic residues and an asparagine residue in the helices 6–8 that are conserved in all isoforms of human anoctamin family form a Ca^2+^ cage that harbors probably two Ca^2+^ atoms. Previously in mutagenesis studies, some of the acidic residues were predicted its engagement for Ca^2+^ binding [106, 118]. This subunit cavity appears to be a pore for ion conduction or phospholipid transport and a Ca^2+^ binding site. Then, how ANO1 is gated by Ca^2+^? One simple model is that upon Ca^2+^ binding, an allosteric change induces an opening of the pore leading to conduction. Then, how do anoctamin genes work as a channel and a scramblase? One good model is ANO6 because it is a scramblase and forms a channel [117]. How does ANO6 work as phospholipid scramblase and a channel activated by Ca^2+^? Recently, Yu and colleagues answered this question [119]. Using a phosphatidyl serine-sensitive fluorescent probe, they could measure Ca^2+^-activated currents and scramblase activity at the same time. Currents are activated slowly 8 min after forming whole cells by high intracellular Ca^2+^ (>20 μM) in cells expressing ANO6. These Ca^2+^-induced currents are coincidental with the scramblase activity. In addition, ANO6 currents show poor selectivity on cations and anions, as if they are leaky currents conducting through large pores [119]. More importantly, constructing various chimeras of ANO6 with ANO1 that does not have scramblase activity, a domain in ANO6 essential for its scramblase activity was searched. A small peptide region spanning only 15 amino acids between TM4 and TM5 of ANO6 is critical for the scramblase activity. When this scramblase domain of ANO6 was replaced with the innate region of ANO1, the ANO1-ANO6-ANO1 chimera gained the scramblase activity [119]. In addition, this chimera exhibits two different currents activated by Ca^2+^, one is a fast-conducting current blocked by MONNA, an ANO1-specific blocker [78], and the other one is a slowly-conducting current that is not blocked by MONNA. Thus, this brilliant study leads to the conclusion that ions flow through the pathway where the phospholipids pass through in ANO6 (Fig. 1). However, because the activation kinetics and ion selectivity of ANO1 and ANO6 currents are quite different and Ca^2+^-induced currents of the ANO1-ANO6-ANO1 chimera have different sensitivity levels to MONNA, Cl^−^-conducting pores may be different from those conduits transporting phospholipids. Therefore, ANO1 may have ion-conducting pores other than the phospholipid transport pathway. However, the latter idea may not be easily accepted because of a report from Jan’s group. Peters and colleagues scanned the basic residues in the 10 TM helices of ANO1 and found that four basic residues that clustered around the opening of the subunit cavity of nhTMEM16 are important for determining ion selectivity of ANO1 [85]. These results strongly indicate that the subunit cavity forms an ion-conducting pore. Because the subunit cavity contains the scramblase domain of ANO6 [119], thus, ions conducting through ANO1 may use the same pathway as phospholipids are transported (Fig. 1). To determine whether an ion-conducting pore in ANO1 uses the same pathway that phospholipids use in ANO6, further studies are needed to be done.

## Anoctamins in nociception

Somatosensory neurons are implicated in touch, proprioception, and pain. DRG neurons extend their axons to the peripheral terminals where nociceptive cues such as heat, cold, mechanical, and chemical stimuli are transduced to electrical signals. CaCCs in DRG neurons are known to be activated by physiological intracellular Ca^2+^, which depolarizes the sensory neurons [6, 95, 98]. ANO1 is expressed mainly in a small diameter of DRG neurons, which are also positive for TRPV1, a nociceptive marker [25]. Surprisingly, ANO1 is known to be activated by noxious heat over ∼44 °C [24, 25], which is slightly over the threshold temperature for pain in human [20, 107]. The activation by heat appears to be direct because ANO1 is activated by heat in the absence of intracellular Ca^2+^ [25]. In addition, the application of heat to DRG neurons isolated from TRPV1-deficient mice depolarizes the neurons, suggesting ANO1’s role for the depolarization. A physiological phenotype was determined with mice deficient of ANO1 in DRG neurons. Heat-activated chloride currents are reduced in DRG neurons from ANO1 conditional knockout mice. More importantly, mice lacking ANO1 in DRG neurons are significantly insensitive to noxious heat, suggesting that ANO1 mediates acute thermal pain [25]. In addition to the heat-evoked acute pain, ANO1 appears to be involved in inflammatory and neuropathic pain. ANO1 conditional knockout mice show a reduction in inflammatory hyperalgesia as well as mechanical allodynia in a neuropathic pain model [61]. Recently, Tominaga and his group reported that TRPV1 and ANO1 interact with each other physically and functionally. Blocking the ANO1 activity significantly reduces the capsaicin-induced pain-related behaviors [104]. Thus, the TRPV1 and ANO1 interaction provides pain-enhancing effects on nociceptors.

A study done by Gamper and his group revealed that bradykinin, an algogen released when tissues are damaged, opens ANO1 via the B_2_ receptor and phospholipase C pathway in nociceptors [64]. Opening of ANO1 causes depolarization of membrane and significantly stimulates action potential firing in DRG neurons [64]. In this study, ANO1 is known to be localized in very close proximity to B_2_ receptor, IP_3_, and endoplasmic reticulum in DRG neurons.

ANO3 (TMEM16C) appears to contribute to nociception. ANO3 is associated with a sodium-activated potassium (Slack) channel [44]. ANO3 is expressed in isolectin B4-positive DRG neurons. Somehow, genetic ablation of ANO3 reduces the expression of Slack channel as well as its currents. In addition, ANO3 overexpression promotes an increase in Slack channel activity. In nociceptors, Slack is responsible for reducing AP firing depending on intracellular sodium concentrations [91]. Thus, because ANO3 enhances Slack activity, ANO3 activity would dampen the excitability of nociceptors. Indeed, ANO3-ablated rats reveal nociceptive hypersensitivity [44].

## ANO1 in smooth muscles

Many research groups have studied CaCC activity in smooth muscle cells from ear, coronary, aortic, and mesenteric arteries and portal vein [3, 31, 55, 59, 82]. Because of the significant role of CaCCs in vascular contractility and proliferation, molecular candidates for CaCCs have been awaited to prove their in vivo vascular functions [23, 113]. Indeed, ANO1 expression and activity were confirmed in smooth muscle cells from various arteries and veins [28, 29, 67, 105]. An ANO1 blocker induces vasorelaxation in murine and human arteries [29]. An active functional role of ANO1 in vascular smooth muscles further came from the study of myogenic response in cerebral arteries. A myogenic response represents a pressure-induced constriction of arteries, which is an innate reflex mechanism that controls local blood flow. Bulley and colleagues found that ANO1 is actively involved in the myogenic response in the cerebral artery [16]. Knockdown of ANO1 suppresses pressure-induced vasoconstriction of the cerebral artery or pressure-induced depolarization of vascular smooth muscle cells [16]. In addition, the ANO1 expression level and its activity are significantly upregulated in various hypertension models [38, 101].

ANO1 is not present in smooth muscle cells in gastrointestinal tracts. However, ANO1 is expressed in the interstitial cells of Cajal, the pacemaker cells of the stomach and intestines [43, 47]. More importantly, smooth muscle contraction in the stomach of ANO1-deficient mouse was markedly attenuated [43, 47].

ANO1 is also expressed in smooth muscle cells and epithelial cells in the airway [46]. In addition, ANO1 expression and activity are increased in airway smooth muscle cells from asthmatic mouse models and human asthmatic patients. Inhibition of ANO1 activity also reduces airway smooth muscle contraction challenged by cholinergic agonists [46]. Thus, these results suggest that ANO1 mediates hypersensitivity in asthmatic airway.

## ANO1 for epithelial Cl^−^ secretion

Epithelium is a tissue that covers surfaces, cavities, or glands of the body. Secretory epithelial cells are necessary for fluid or electrolyte secretion in various biological processes [83, 110]. Especially, Cl^−^ flow in those cells plays a crucial role in determining the way of fluid or electrolyte secretion [7]. As Cl^−^ is important for transepithelial secretion, CaCC activity was observed in numerous transepithelial tissues, including airway epithelium, salivary glands, pancreatic ductal cells, and intestinal epithelium [39]. In many transport epithelia, CaCCs are considered as an alternative pathway of Cl^−^ secretion for CFTR. Thus, the role of CaCC in epithelial secretion was vigorously studied. Therefore, ANO1 became a primary target for the study of Cl^−^ secretion in these tissues.

In airway, transport of Cl^−^ ions across the airway epithelium is required for the protecting mechanism against microbial infection because the Cl^−^ secretion accompanied with water secretion induces hydration of the airway epithelium. ANO1 is expressed in mouse and human airway epithelial cells [43, 46, 96]. In a previous study, it was known that T helper 2 (Th2) cytokines such as IL-4 and IL-13 upregulate Ca^2+^-dependent Cl^−^ secretion in the human bronchial epithelium [40]. Based on this information, Caputo and colleagues were able to clone *Ano1* from bronchial epithelial cells using the differential hybridization after treating the epithelial cells with IL-4 [18]. Thus, an ANO1’s role for Cl^−^ secretion in the bronchial epithelium was expected. Indeed, knockdown of ANO1 by small interfering RNA (siRNA) treatment significantly inhibited the Ca^2+^-dependent Cl^−^ secretion in the IL-4-treated epithelial cells [18]. Furthermore, ANO1 expression was strongly upregulated in airway epithelial cells after stimulation of IL-4 and IL-13, a condition that mimics asthmatic or allergic inflammation [46, 96]. The increased expression of ANO1 was also found in airway epithelial cells of Th2 cytokines-high human asthma patients [46]. In normal condition, ANO1 was found to be minimally expressed in goblet cells which were co-expressed with mucin 5 AC, a marker for goblet cell. However, the expression of ANO1 was strongly enhanced in mucin 5AC-positive goblet cells of ovalbumin-challenged mice or asthmatic patients [46]. In addition, pyocyanin, a major virulence factor of *Pseudomonas aeruginosa*, or bacterial supernatants isolated from *P. aeruginosa* upregulated ANO1 expression in mucin 5AC-positive goblet cells and Ca^2+^-dependent Cl^−^ secretion in bronchial epithelial cells [17]. Thus, ANO1 appears to be important for Cl^−^ secretion in the bronchial epithelium in pathologic conditions.

ANO1 is also observed in the intestinal epithelium which is responsible for absorptive and secretory functions in intestines. ANO1 is expressed in epithelial cells of the small intestine and colon [80, 93]. In addition, carbachol-induced Cl^−^ secretion in the distal colon was lacking in ANO1 knockout mice [80]. Schreiber and colleagues constructed conditional knockout mice that lack an ANO1 gene in the intestinal epithelium and found that the conditional knockout in the colon suppressed Ca^2+^-dependent secretion of Cl^−^ in the colon [93]. In contrast, ANO1 blockers minimally inhibited the Ca^2+^-dependent Cl^−^ secretion in colonic cell lines [74]. Interestingly, the acute exposure of epidermal growth factor potentiated Ca^2+^-dependent Cl^−^ secretion as well as elevated ANO1 expression in colonic epithelial cells [73]. A rotavirus toxin, NSP4, known for inducing diarrhea in infants also causes Ca^2+^-dependent transepithelial secretion. When a synthetic NSP4 peptide was treated on ANO1-transfected HEK293 cells, it induced the Ca^2+^-dependent Cl^−^ secretion by activating ANO1 [81]. Thus, it is clear that ANO1 plays a role in Cl^−^ secretion in intestines.

Activation of the CaCCs in the salivary gland triggers the saliva secretion [70]. In line with this, ANO1 immunoreactivity was found in the apical membrane of the mouse salivary gland [88, 116]. In addition, knockdown of ANO1 by siRNA treatment reduces salivary secretion induced by pilocarpine [116]. Two groups found a strong phenotype in ANO1-ablated mice. Systemic knockout of ANO1 abolished a Ca^2+^-activated Cl^−^ current in submandibular acinar cells as well as fluid secretion in the submandibular gland [88]. Moreover, a targeted deletion of ANO1 at the salivary gland completely eliminated Ca^2+^-dependent Cl^−^ current and salivary secretion [19]. Thus, it is clear that ANO1 is critical for the Cl^−^ efflux and salivation in the salivary gland.

ANO1 appears to be essential for pancreatic β cell function [26]. Most of the pancreatic islet cells express ANO1. Because fluctuation of membrane potential along with action potential firings is important for insulin secretion, the effect of ANO1 blockade on glucose-induced membrane depolarization was determined by Crutzen and colleagues [26]. Surprisingly, glucose-induced insulin secretion as well as glucose-induced membrane potential oscillation are abolished by an ANO1-specific blocker, T-AO1, in pancreatic islets or β cells [26]. Thus, this study clearly suggests that ANO1 is critical for the glucose-induced membrane fluctuation in β cell that is necessary for insulin secretion. In line with this, ANO1 haploinsufficiency impairs insulin secretion in mice [115].

ANO1 is also expressed in Madin-Darby canine kidney (MDCK) epithelial cell line which is widely used for studying the growth of kidney cysts, which suggests a role in renal function [15]. Knockdown of ANO1 using siRNA inhibits UTP-induced Cl^−^ secretion in MDCK cells. Similarly, the knockdown of ANO1 or ANO1 inhibitor suppresses cyst growth in the model of kidney cyst growth [15]. Besides the cyst growth, ANO1 mediates acid secretion and protein reabsorption in the proximal tubules of the kidney. Faria and colleagues confirmed a strong expression in the proximal tubule epithelium in the mouse and human kidney [37]. Mice lacking ANO1 in tubular epithelial cells elicit proteinuria and a reduction in proton secretion [37]. Thus, ANO1 plays a role in Cl^−^ secretion in the renal proximal tubule, which is required for H^+^ secretion to reabsorb HCO_3_^−^ in the renal tubules.

Several studies also suggest the secretory function of ANO1 in the biliary and sweat gland epithelia. In the mouse and human biliary epithelium, bile flow increases Cl^−^ currents [33]. Silencing of ANO1 by siRNA treatment significantly attenuated the flow-stimulated Cl^−^ currents in human biliary epithelial cells [33]. In addition, in human NCL-SG3 sweat gland cells, ANO1 was responsible for Ca^2+^-dependent Cl^−^ secretion [35]. ANO1 transcripts were also identified in native human sweat gland tissues [35].

## ANO1 function in tumorigenesis and proliferation

The growing evidence of the role of ANO1 in cancer has been suggested before its molecular identity was discovered. *FLJ10261* gene (now known as *Ano1*) was identified in the *CCND1-EMS1* locus on human chromosome 11q13 which is frequently amplified in various types of tumors [52]. Structural analysis predicted that FLJ10261 protein possessed eight TM domains possibly functioning as an ion transporter [52]. Since the *FLJ10261* gene was found to be uniformly expressed with a high level of gastrointestinal stromal tumors (GISTs) thereby being named *DOG1* (discovered on GIST1) [111], ANO1/DOG1 has been emerging as a potential diagnostic marker for GIST [36, 48, 62, 71, 77].

Although ANO1 is found to be widely expressed in various tissues including the secretory epithelium [49], *Ano1* has been found to be upregulated in numerous carcinomas including head and neck squamous cell carcinoma (HNSCC) [5, 30, 34], lung cancer [50], breast cancer [13, 112], colorectal cancer [100], pancreatic ductal adenocarcinoma [92], gastrointestinal stromal tumor [111], esophageal squamous cell carcinoma [51, 97], chondroblastoma [2], salivary gland tumor (designated as *ORAOV2*, oral cancer overexpressed 2) [22], oral cancer (designated as *TAOS1*, tumor-amplified and overexpressed sequence 1) [42, 63], uterine leiomyosarcoma [90], glioma [65], and prostate cancer [66]. The ANO1 expression in various tumors is summarized in Table 1.

**Table 1:** Anoctamin 1 function in cancer

Cancer type	Main results	Effects of ANO1 inhibition	Experimental system	References
Head and neck squamous cell carcinoma (HNSCC)	HPV(−) HNSCC expresses more Ano1 than HPV+, DNA methylation	Decreased tumor size Cell growth inhibition	In vitro Patient TMA	[30]
HNSCC	Promoted tumor growth and proliferation Decreased patient survival Induced phospho-ERK1/2 and cyclin D1	Growth inhibition of tumor xenografts Decreased proliferation	In vitro tumor xenograft (mouse) Kaplan–Meier survival analysis	[34]
HNSCC	ANO1 expression stimulates cell migration, invasion, adhesion, spreading, and detachment	Decreased movement	Patient sample Cell line	[5]
HNSCC	Poor survival, cell volume regulation and cell migration	No effect on cell proliferation	TMA In vitro Patient survival assay	[89]
Lung adenocarcinoma	Lung cancer progression	Decreased tumor growth and invasion	In vitro	[50]
Breast cancer	Potential marker for good prognosis in PR+ or HER2− patients following tamoxifen treatment		Patient sample	[112]
Breast cancer	Breast cancer progression by EGFR-CaMKII signaling activation	Decreased tumor growth, reduced proliferation, induced apoptosis, reduced EGFR-CaMKII, and reduced AKT, SRC, and ERK	In vitro In vivo (mouse)	[13]
Colorectal cancer (CRC)		Decreased cell growth, migration, and invasion Decreased MEK-ERK1/2 and cyclin D1 expression	In vitro	[100]
Pancreatic ductal adenocarcinoma (PDAC)	Functional ANO1 upregulation	Reduced cell migration, unaffected cell proliferation	In vitro	[92]
Chondroblastoma (bone tumor)	Immunohistochemical marker for chondroblastoma		Patient sample	[2]
Salivary gland tumor	Diagnosis of acinic cell carcinoma		Tissue sample	[22]
Esophageal squamous cell carcinoma (ESCC)	ESCC biomarker		Whole-genome DNA microarray TMA	[51]
ESCC		Reduced proliferation	Array CGH on ESCC	[97]
Oral cancer	Physical mapping at human chromosome 11q13		In vitro	[42]
Oral squamous cell carcinoma (OSCC)	Promoted migration	Reduced migration	Patient sample	[63]
Uterine leiomyosarcomas	Lymph node metastasis		In vitro	[90]
Glioma	Activation of NF-κB	Reduced cell proliferation, migration, and invasion	Tumor tissue In vitro	[65]
Prostate carcinoma	Correlation with the TNM stage and Gleason score	Reduced proliferation, metastasis, and invasion Inhibition of tumor growth and survival	In vivo (mouse) In vitro	[66]

*Ano1* gene amplification in tumors showed a significant correlation with poor survival rate in cancer patients [13, 34, 89], positive correlation with tumor grade [66], the increase in cell migration [5], and tumor growth or metastasis [97]. Thus, ANO1 is highly associated with tumor and its progression. Then, what is the role of ANO1 in tumorigenesis? Many investigations have highlighted signaling pathways of ANO1-mediated tumor progression, which requires multiple cellular events including cell proliferation, migration/invasion, tumor growth, and metastasis in vivo. Duvvuri and colleagues found the mitogen-activated protein kinase (MAPK) activation during ANO1-mediated tumor progression [34]. ANO1 overexpression induces tumor growth in vivo and cell proliferation by activating extracellular signal-regulated kinase (ERK)1/2 via the ras-raf-MEK-ERK pathway and cyclin D1, but not activating AKT [34]. Knockdown of ERK or specific inhibitors of MEK/ERK blocks the ANO1-mediated cell proliferation. In addition, ANO1 knockdown abrogated cell proliferation in vitro and tumor growth of HNSCC tumor xenografts, which parallels with the cell cycle arrest at G_1_/S phase transition [34].

ANO1 has been also suggested as a regulator of epidermal growth factor receptor (EGFR) signaling. EGFR, a receptor tyrosine kinase, undergoes dimerization upon ligand binding and then phosphorylation of tyrosine residues, which leads to the initiation of the MAPK or PI3K-AKT pathway. EGFR has been implicated to be ubiquitously overexpressed in HNSCC [4]. The phosphorylation of EGFR exhibits poor prognosis such as metastatic lymph node and early relapse in HNSCC patients [41]. Knockdown of ANO1 diminishes cell viability and induces apoptosis, indicating pro-survival and anti-apoptotic function of ANO1 [13]. ANO1 knockdown strongly suppresses EGFR phosphorylation due to a reduction in autocrine EGFR ligand secretion. ANO1 inhibition subsequently leads to the reduction in phosphorylation of ERK1/2, AKT and v-src in breast cancer cells, HNSCCs, and esophageal squamous carcinoma cell lines [13]. ANO1 inhibition also blocks calcium/calmodulin-dependent protein kinase II (CaMKII) phosphorylation. Thus, these results imply that ANO1 regulates cell viability via EGFR-AKT/SRC/MAPK pathway and calcium-dependent CaMKII signaling. Furthermore, ANO1 was identified to interact and form a functional complex with EGFR in HNSCC to regulate cell proliferation. Thus, while ANO1 expression stabilized EGFR, EGFR signaling upregulated ANO1 protein level, which establishes positive cooperation between ANO1 and EGFR [9].

Additionally, ANO1 was reported to associate physically with ezrin-radixin-moesin (ERM) [84]. ERM proteins cross-link between plasma membrane and actin filaments involving many cellular events including cytoskeletal organization, cell division, and cell migration/invasion [109]. In normal state, ERM proteins are suppressed by an intramolecular head-to-tail association. In abnormal state, ERM becomes activated via phosphorylation by many ligands including EGF and platelet-derived growth factor (PDGF) [8]. Sphingosine-1-phosphate was suggested to mediate EGF-induced ERM phosphorylation leading to cancer cell invasion [1, 79]. Interestingly, an ANO1 current was reduced by moesin knockdown [84], indicating ANO1 regulation by ERM stoichiometry. Thus, an ANO1-ERM interaction might provide a clue to the role of ANO1 in EGF-driven tumor cell migration and invasion.

How can ANO1 be highly amplified in tumors? Transcriptional regulation occurring in the ANO1 promoter region could provide a clue to the aberrant ANO1 expression in cancer. Indeed, the promoter region contains putative binding sites for an androgen response element (ARE), which allow testosterone-induced ANO1 upregulation in prostate cells [21]. Signal transducer and activator of transcription 6 (STAT6) binding site is also found in the human ANO1 promoter region, leading to IL-4-induced ANO1 upregulation [69]. In addition, ANO1 expression may be tightly regulated by epigenetic factors. For example, human papilloma virus (HPV)-negative tumors express a higher level of ANO1 than HPV-positive ones [30]. A methylation level within the ANO1 promoter region was lower in HPV-negative tumors than that in HPV-positive tumors. In addition, histone deacetylase (HDAC) inhibitors downregulated ANO1 expression and its activity in prostate or breast cancer cell lines, resulting in suppression of cancer cell viability [68].

The role of ANO1 in promoting cell proliferation seems to be not confined in the tumor microenvironment. In normal head and neck tissues, ANO1 knockout mice showed decreased cyclin D1 expression as compared to the wild type, suggesting that ANO1 influences the basal level of cell proliferation [34]. Recently, Cha and colleagues demonstrated that ANO1 is essential for benign prostatic hyperplasia (BPH) [21]. *Ano1* was highly amplified in testosterone-treated prostate epithelia [21]. The presence of androgen response element in the ANO1 promoter region supports the transcriptional control of ANO1 by testosterone [21]. More importantly, inhibition of ANO1 resulted in the suppression of cell proliferation and prostate enlargement. Thus, ANO1 activity appears to control the testosterone-induced cell proliferation [21].

It remains unclear whether ANO1-mediated tumor progression or cell proliferation is merely due to the increased mRNA level of ANO1 or due to the augmented channel activity. Surprisingly, inhibition of ANO1 with blockers or mutation of ANO1 in putative pore region suppresses growth promotion [13, 21, 34]. Thus, it is remarkable that the functional channel activity of ANO1 is required for cell viability, promotion of tumor growth, and cell proliferation. On the contrary, whereas several compounds that are known to inhibit ANO1 activity fail to inhibit ANO1-dependent cell proliferation, CaCCinh-A01, which promotes ANO1 degradation, efficiently inhibits cell proliferation [10]. This result implies that the protein level of ANO1 is more requisite for ANO1-induced cell proliferation rather than ANO1 channel function. Because the previous investigations on ANO1 in cancer have a limit to measure channel activity, therefore, we cannot differentiate between overall increase in channel expression and increased channel activity in tumors. Future experiments are needed to be done on examining channel activity in tumors compared to normal cells. The other possible cause is a change in intracellular Cl^−^ concentration [Cl^−^] in cancer cells. As ANO1 is a channel embedded within the plasma membrane conducting Cl^−^, opening of ANO1 results in change in intracellular [Cl^−^] or membrane potentials. This change in intracellular [Cl^−^] or membrane potentials may subsequently activate oncogenic signaling cascades such as MAPK or AKT. However, which signaling pathway is induced by the activation of ANO1 in cancer cells remains still unknown. Further work will be needed to clarify this signaling cascade.

## References

[1] Adada MM, Canals D, Jeong N, Kelkar AD, Hernandez-Corbacho M, Pulkoski-Gross MJ, Donaldson JC, Hannun YA, Obeid LM (2015). Intracellular sphingosine kinase 2-derived sphingosine-1-phosphate mediates epidermal growth factor-induced ezrin-radixin-moesin phosphorylation and cancer cell invasion. FASEB J.

[2] Akpalo H, Lange C, Zustin J (2012). Discovered on gastrointestinal stromal tumour 1 (DOG1): a useful immunohistochemical marker for diagnosing chondroblastoma. Histopathology.

[3] Amedee T, Large WA, Wang Q (1990). Characteristics of chloride currents activated by noradrenaline in rabbit ear artery cells. J Physiol.

[4] Ang KK, Berkey BA, Tu X, Zhang HZ, Katz R, Hammond EH, Fu KK, Milas L (2002). Impact of epidermal growth factor receptor expression on survival and pattern of relapse in patients with advanced head and neck carcinoma. Cancer Res.

[5] Ayoub C, Wasylyk C, Li Y, Thomas E, Marisa L, Robe A, Roux M, Abecassis J, de Reynies A, Wasylyk B (2010). ANO1 amplification and expression in HNSCC with a high propensity for future distant metastasis and its functions in HNSCC cell lines. Brit J Cancer.

[6] Bader CR, Bertrand D, Schlichter R (1987). Calcium-activated chloride current in cultured sensory and parasympathetic quail neurones. J Physiol.

[7] Barrett KE, Keely SJ (2000). Chloride secretion by the intestinal epithelium: molecular basis and regulatory aspects. Ann Rev Physiol.

[8] Baumgartner M, Sillman AL, Blackwood EM, Srivastava J, Madson N, Schilling JW, Wright JH, Barber DL (2006). The Nck-interacting kinase phosphorylates ERM proteins for formation of lamellipodium by growth factors. Proc Natl Acad Sci U S A.

[9] Bill A, Gutierrez A, Kulkarni S, Kemp C, Bonenfant D, Voshol H, Duvvuri U, Gaither LA (2015). ANO1 interacts with EGFR and correlates with sensitivity to EGFR-targeting therapy in head and neck cancer. Oncotarget.

[10] Bill A, Hall ML, Borawski J, Hodgson C, Jenkins J, Piechon P, Popa O, Rothwell C, Tranter P, Tria S, Wagner T, Whitehead L, Gaither LA (2014). Small molecule-facilitated degradation of ANO1 protein: a new targeting approach for anticancer therapeutics. J Biol Chem.

[11] Billig GM, Pal B, Fidzinski P, Jentsch TJ (2011). Ca^2+^-activated Cl^−^ currents are dispensable for olfaction. Nat Neurosci.

[12] Bolduc V, Marlow G, Boycott KM, Saleki K, Inoue H, Kroon J, Itakura M, Robitaille Y, Parent L, Baas F, Mizuta K, Kamata N, Richard I, Linssen WH, Mahjneh I, de Visser M, Bashir R, Brais B (2010). Recessive mutations in the putative calcium-activated chloride channel anoctamin 5 cause proximal LGMD2L and distal MMD3 muscular dystrophies. Am J Hum Genet.

[13] Britschgi A, Bill A, Brinkhaus H, Rothwell C, Clay I, Duss S, Rebhan M, Raman P, Guy CT, Wetzel K, George E, Popa MO, Lilley S, Choudhury H, Gosling M, Wang L, Fitzgerald S, Borawski J, Baffoe J, Labow M, Gaither LA, Bentires-Alj M (2013). Calcium-activated chloride channel ANO1 promotes breast cancer progression by activating EGFR and CAMK signaling. Proc Natl Acad Sci U S A.

[14] Brunner JD, Lim NK, Schenck S, Duerst A, Dutzler R (2014). X-ray structure of a calcium-activated TMEM16 lipid scramblase. Nature.

[15] Buchholz B, Faria D, Schley G, Schreiber R, Eckardt KU, Kunzelmann K (2014). Anoctamin 1 induces calcium-activated chloride secretion and proliferation of renal cyst-forming epithelial cells. Kidney Int.

[16] Bulley S, Neeb ZP, Burris SK, Bannister JP, Thomas-Gatewood CM, Jangsangthong W, Jaggar JH (2012). TMEM16A/ANO1 channels contribute to the myogenic response in cerebral arteries. Circ Res.

[17] Caci E, Scudieri P, Di Carlo E, Morelli P, Bruno S, De Fino I, Bragonzi A, Gianotti A, Sondo E, Ferrera L, Palleschi A, Santambrogio L, Ravazzolo R, Galietta LJ (2015). Upregulation of TMEM16A protein in bronchial epithelial cells by bacterial pyocyanin. PLoS One.

[18] Caputo A, Caci E, Ferrera L, Pedemonte N, Barsanti C, Sondo E, Pfeffer U, Ravazzolo R, Zegarra-Moran O, Galietta LJ (2008). TMEM16A, a membrane protein associated with calcium-dependent chloride channel activity. Science.

[19] Catalan MA, Kondo Y, Pena-Munzenmayer G, Jaramillo Y, Liu F, Choi S, Crandall E, Borok Z, Flodby P, Shull GE, Melvin JE (2015). A fluid secretion pathway unmasked by acinar-specific Tmem16A gene ablation in the adult mouse salivary gland. Proc Natl Acad Sci U S A.

[20] Caterina MJ, Schumacher MA, Tominaga M, Rosen TA, Levine JD, Julius D (1997). The capsaicin receptor: a heat-activated ion channel in the pain pathway. Nature.

[21] Cha JY, Wee J, Jung J, Jang Y, Lee B, Hong GS, Chang BC, Choi YL, Shin YK, Min HY, Lee HY, Na TY, Lee MO, Oh U (2015). Anoctamin 1 (TMEM16A) is essential for testosterone-induced prostate hyperplasia. Proc Natl Acad Sci U S A.

[22] Chenevert J, Duvvuri U, Chiosea S, Dacic S, Cieply K, Kim J, Shiwarski D, Seethala RR (2012). DOG1: a novel marker of salivary acinar and intercalated duct differentiation. Mod Pathol.

[23] Cheng G, Kim MJ, Jia G, Agrawal DK (2007). Involvement of chloride channels in IGF-I-induced proliferation of porcine arterial smooth muscle cells. Cardiovasc Res.

[24] Cho H, Oh U (2013). Anoctamin 1 mediates thermal pain as a heat sensor. Curr Neuropharmacol.

[25] Cho H, Yang YD, Lee J, Lee B, Kim T, Jang Y, Back SK, Na HS, Harfe BD, Wang F, Raouf R, Wood JN, Oh U (2012). The calcium-activated chloride channel anoctamin 1 acts as a heat sensor in nociceptive neurons. Nature Neurosci.

[26] Crutzen R, Virreira M, Markadieu N, Shlyonsky V, Sener A, Malaisse WJ, Beauwens R, Boom A, and Golstein PE (2015) Anoctamin 1 (Ano1) is required for glucose-induced membrane potential oscillations and insulin secretion by murine beta-cells. Pflugers Arch 10.1007/s00424-015-1758-5 PMC4792454 26582426

[27] Currie KP, Wootton JF, Scott RH (1995). Activation of Ca(2+)-dependent Cl- currents in cultured rat sensory neurones by flash photolysis of DM-nitrophen. J Physiol.

[28] Davis AJ, Forrest AS, Jepps TA, Valencik ML, Wiwchar M, Singer CA, Sones WR, Greenwood IA, Leblanc N (2010). Expression profile and protein translation of TMEM16A in murine smooth muscle. Am J Physiol Cell Physiol.

[29] Davis AJ, Shi J, Pritchard HA, Chadha PS, Leblanc N, Vasilikostas G, Yao Z, Verkman AS, Albert AP, Greenwood IA (2012). Potent vasorelaxant activity of the TMEM16A inhibitor T16A(inh)-A01. Br J Pharmacol.

[30] Dixit R, Kemp C, Kulich S, Seethala R, Chiosea S, Ling S, Ha PK, Duvvuri U (2015). TMEM16A/ANO1 is differentially expressed in HPV-negative versus HPV-positive head and neck squamous cell carcinoma through promoter methylation. Sci Rep.

[31] Droogmans G, Callewaert G, Declerck I, Casteels R (1991). ATP-induced Ca^2+^ release and Cl^−^ current in cultured smooth muscle cells from pig aorta. J Physiol.

[32] Duran C, Qu Z, Osunkoya AO, Cui Y, Hartzell HC (2012). ANOs 3-7 in the anoctamin/Tmem16 Cl- channel family are intracellular proteins. Am J Physiol Cell Physiol.

[33] Dutta AK, Woo K, Khimji AK, Kresge C, Feranchak AP (2013). Mechanosensitive Cl- secretion in biliary epithelium mediated through TMEM16A. Am J Physiol Gastrointest Liver Physiol.

[34] Duvvuri U, Shiwarski DJ, Xiao D, Bertrand C, Huang X, Edinger RS, Rock JR, Harfe BD, Henson BJ, Kunzelmann K, Schreiber R, Seethala RS, Egloff AM, Chen X, Lui VW, Grandis JR, Gollin SM (2012). TMEM16A induces MAPK and contributes directly to tumorigenesis and cancer progression. Cancer Res.

[35] Ertongur-Fauth T, Hochheimer A, Buescher JM, Rapprich S, Krohn M (2014). A novel TMEM16A splice variant lacking the dimerization domain contributes to calcium-activated chloride secretion in human sweat gland epithelial cells. Exp Dermatol.

[36] Espinosa I, Lee CH, Kim MK, Rouse BT, Subramanian S, Montgomery K, Varma S, Corless CL, Heinrich MC, Smith KS, Wang Z, Rubin B, Nielsen TO, Seitz RS, Ross DT, West RB, Cleary ML, van de Rijn M (2008). A novel monoclonal antibody against DOG1 is a sensitive and specific marker for gastrointestinal stromal tumors. Am J Surg Path.

[37] Faria D, Rock JR, Romao AM, Schweda F, Bandulik S, Witzgall R, Schlatter E, Heitzmann D, Pavenstadt H, Herrmann E, Kunzelmann K, Schreiber R (2014). The calcium-activated chloride channel anoctamin 1 contributes to the regulation of renal function. Kidney Int.

[38] Forrest AS, Joyce TC, Huebner ML, Ayon RJ, Wiwchar M, Joyce J, Freitas N, Davis AJ, Ye L, Duan DD, Singer CA, Valencik ML, Greenwood IA, Leblanc N (2012). Increased TMEM16A-encoded calcium-activated chloride channel activity is associated with pulmonary hypertension. Am J Physiol Cell Physiol.

[39] Fuller CM (2002). Calcium-activated chloride channels.

[40] Galietta LJ, Pagesy P, Folli C, Caci E, Romio L, Costes B, Nicolis E, Cabrini G, Goossens M, Ravazzolo R, Zegarra-Moran O (2002). IL-4 is a potent modulator of ion transport in the human bronchial epithelium in vitro. J Immunol.

[41] Hama T, Yuza Y, Saito Y, Ou J, Kondo S, Okabe M, Yamada H, Kato T, Moriyama H, Kurihara S, Urashima M (2009). Prognostic significance of epidermal growth factor receptor phosphorylation and mutation in head and neck squamous cell carcinoma. Oncologist.

[42] Huang X, Gollin SM, Raja S, Godfrey TE (2002). High-resolution mapping of the 11q13 amplicon and identification of a gene, TAOS1, that is amplified and overexpressed in oral cancer cells. Proc Natl Acad Sci U S A.

[43] Huang F, Rock JR, Harfe BD, Cheng T, Huang X, Jan YN, Jan LY (2009). Studies on expression and function of the TMEM16A calcium-activated chloride channel. Proc Natl Acad Sci U S A.

[44] Huang F, Wang X, Ostertag EM, Nuwal T, Huang B, Jan YN, Basbaum AI, Jan LY (2013). TMEM16C facilitates Na(+)-activated K+ currents in rat sensory neurons and regulates pain processing. Nat Neurosci.

[45] Huang WC, Xiao S, Huang F, Harfe BD, Jan YN, Jan LY (2012). Calcium-activated chloride channels (CaCCs) regulate action potential and synaptic response in hippocampal neurons. Neuron.

[46] Huang F, Zhang H, Wu M, Yang H, Kudo M, Peters CJ, Woodruff PG, Solberg OD, Donne ML, Huang X, Sheppard D, Fahy JV, Wolters PJ, Hogan BL, Finkbeiner WE, Li M, Jan YN, Jan LY, Rock JR (2012). Calcium-activated chloride channel TMEM16A modulates mucin secretion and airway smooth muscle contraction. Proc Natl Acad Sci U S A.

[47] Hwang SJ, Blair PJ, Britton FC, O’Driscoll KE, Hennig G, Bayguinov YR, Rock JR, Harfe BD, Sanders KM, Ward SM (2009). Expression of anoctamin 1/TMEM16A by interstitial cells of Cajal is fundamental for slow wave activity in gastrointestinal muscles. J Physiol.

[48] Hwang DG, Qian X, Hornick JL (2011). DOG1 antibody is a highly sensitive and specific marker for gastrointestinal stromal tumors in cytology cell blocks. Am J Clin Pathol.

[49] Jang Y, and Oh U (2014) Anoctamin 1 in secretory epithelia. Cell Calcium 10.1016/j.ceca.2014.02.006 24636668

[50] Jia L, Liu W, Guan L, Lu M, Wang K (2015). Inhibition of calcium-activated chloride channel ANO1/TMEM16A suppresses tumor growth and invasion in human lung cancer. PLoS One.

[51] Kashyap MK, Marimuthu A, Kishore CJ, Peri S, Keerthikumar S, Prasad TS, Mahmood R, Rao S, Ranganathan P, Sanjeeviah RC, Vijayakumar M, Kumar KV, Montgomery EA, Kumar RV, Pandey A (2009). Genomewide mRNA profiling of esophageal squamous cell carcinoma for identification of cancer biomarkers. Cancer Biol Ther.

[52] Katoh M, Katoh M (2003). FLJ10261 gene, located within the CCND1-EMS1 locus on human chromosome 11q13, encodes the eight-transmembrane protein homologous to C12orf3, C11orf25 and FLJ34272 gene products. Int J Oncol.

[53] Kleene SJ (1997). High-gain, low-noise amplification in olfactory transduction. Biophys J.

[54] Kleene SJ, Gesteland RC (1991). Calcium-activated chloride conductance in frog olfactory cilia. J Neurosci.

[55] Klockner U (1993). Intracellular calcium ions activate a low-conductance chloride channel in smooth-muscle cells isolated from human mesenteric artery. Pflugers Arch.

[56] Kunzelmann K, Tian Y, Martins JR, Faria D, Kongsuphol P, Ousingsawat J, Thevenod F, Roussa E, Rock J, Schreiber R (2011). Anoctamins. Pflugers Arch.

[57] Kuruma A, Hartzell HC (1999). Dynamics of calcium regulation of chloride currents in Xenopus oocytes. Am J Physiol.

[58] Kuruma A, Hartzell HC (2000). Bimodal control of a Ca(2+)-activated Cl(-) channel by different Ca(2+) signals. J Gen Physiol.

[59] Lamb FS, Volk KA, Shibata EF (1994). Calcium-activated chloride current in rabbit coronary artery myocytes. Circ Res.

[60] Large WA, Wang Q (1996). Characteristics and physiological role of the Ca(2+)-activated Cl- conductance in smooth muscle. Am J Physiol.

[61] Lee B, Cho H, Jung J, Yang YD, Yang DJ, Oh U (2014). Anoctamin 1 contributes to inflammatory and nerve-injury induced hypersensitivity. Mol Pain.

[62] Lee CH, Liang CW, Espinosa I (2010). The utility of discovered on gastrointestinal stromal tumor 1 (DOG1) antibody in surgical pathology-the GIST of it. Adv Anat Pathol.

[63] Li Y, Zhang J, Hong S (2014). ANO1 as a marker of oral squamous cell carcinoma and silencing ANO1 suppresses migration of human SCC-25 cells. Med Oral, Pathol Oral Cir Bucal.

[64] Liu B, Linley JE, Du X, Zhang X, Ooi L, Zhang H, Gamper N (2010). The acute nociceptive signals induced by bradykinin in rat sensory neurons are mediated by inhibition of M-type K+ channels and activation of Ca2+-activated Cl- channels. J Clin Invest.

[65] Liu J, Liu Y, Ren Y, Kang L, Zhang L (2014). Transmembrane protein with unknown function 16A overexpression promotes glioma formation through the nuclear factor-kappaB signaling pathway. Mol Med Rep.

[66] Liu W, Lu M, Liu B, Huang Y, Wang K (2012). Inhibition of Ca(2+)-activated Cl(-) channel ANO1/TMEM16A expression suppresses tumor growth and invasiveness in human prostate carcinoma. Cancer Lett.

[67] Manoury B, Tamuleviciute A, Tammaro P (2010). TMEM16A/anoctamin 1 protein mediates calcium-activated chloride currents in pulmonary arterial smooth muscle cells. J Physiol.

[68] Matsuba S, Niwa S, Muraki K, Kanatsuka S, Nakazono Y, Hatano N, Fujii M, Zhan P, Suzuki T, Ohya S (2014). Downregulation of Ca2+-activated Cl- channel TMEM16A by the inhibition of histone deacetylase in TMEM16A-expressing cancer cells. J Pharmacol Exp Ther.

[69] Mazzone A, Gibbons SJ, Bernard CE, Nowsheen S, Middha S, Almada LL, Ordog T, Kendrick ML, Reid Lombardo KM, Shen KR, Galietta LJ, Fernandez-Zapico ME, Farrugia G (2015). Identification and characterization of a novel promoter for the human ANO1 gene regulated by the transcription factor signal transducer and activator of transcription 6 (STAT6). FASEB J.

[70] Melvin JE, Arreola J, Nehrke K, Begenisich T, Fuller CM (2002). Ca2+-activated Cl- current in salivary and lacrimal glands. Calcium-activated chloride channels.

[71] Miettinen M, Wang ZF, Lasota J (2009). DOG1 antibody in the differential diagnosis of gastrointestinal stromal tumors: a study of 1840 cases. Am J Surg Pathol.

[72] Mizuta K, Tsutsumi S, Inoue H, Sakamoto Y, Miyatake K, Miyawaki K, Noji S, Kamata N, Itakura M (2007). Molecular characterization of GDD1/TMEM16E, the gene product responsible for autosomal dominant gnathodiaphyseal dysplasia. Biochem Biophys Res Commun.

[73] Mroz MS, Keely SJ (2012). Epidermal growth factor chronically upregulates Ca(2+)-dependent Cl(-) conductance and TMEM16A expression in intestinal epithelial cells. J Physiol.

[74] Namkung W, Phuan PW, Verkman AS (2011). TMEM16A inhibitors reveal TMEM16A as a minor component of calcium-activated chloride channel conductance in airway and intestinal epithelial cells. J Biol Chem.

[75] Nilius B, Droogmans G (2001). Ion channels and their functional role in vascular endothelium. Physiol Rev.

[76] Nilius B, Prenen J, Szucs G, Wei L, Tanzi F, Voets T, Droogmans G (1997). Calcium-activated chloride channels in bovine pulmonary artery endothelial cells. J Physiol.

[77] Novelli M, Rossi S, Rodriguez-Justo M, Taniere P, Seddon B, Toffolatti L, Sartor C, Hogendoorn PC, Sciot R, Van Glabbeke M, Verweij J, Blay JY, Hohenberger P, Flanagan A, Dei Tos AP (2010). DOG1 and CD117 are the antibodies of choice in the diagnosis of gastrointestinal stromal tumours. Histopathology.

[78] Oh SJ, Hwang SJ, Jung J, Yu K, Kim J, Choi JY, Hartzell HC, Roh EJ, Lee CJ (2013). MONNA, a potent and selective blocker for transmembrane protein with unknown function 16/anoctamin-1. Mol Pharmacol.

[79] Orr Gandy KA, Adada M, Canals D, Carroll B, Roddy P, Hannun YA, Obeid LM (2013). Epidermal growth factor-induced cellular invasion requires sphingosine-1-phosphate/sphingosine-1-phosphate 2 receptor-mediated ezrin activation. FASEB J.

[80] Ousingsawat J, Martins JR, Schreiber R, Rock JR, Harfe BD, Kunzelmann K (2009). Loss of TMEM16A causes a defect in epithelial Ca2+-dependent chloride transport. J Biol Chem.

[81] Ousingsawat J, Mirza M, Tian Y, Roussa E, Schreiber R, Cook DI, Kunzelmann K (2011). Rotavirus toxin NSP4 induces diarrhea by activation of TMEM16A and inhibition of Na+ absorption. Pflugers Arch.

[82] Pacaud P, Loirand G, Gregoire G, Mironneau C, Mironneau J (1992). Calcium-dependence of the calcium-activated chloride current in smooth muscle cells of rat portal vein. Pflugers Arch.

[83] Pacha J (2000). Development of intestinal transport function in mammals. Physiol Rev.

[84] Perez-Cornejo P, Gokhale A, Duran C, Cui Y, Xiao Q, Hartzell HC, Faundez V (2012). Anoctamin 1 (Tmem16A) Ca2+-activated chloride channel stoichiometrically interacts with an ezrin-radixin-moesin network. Proc Natl Acad Sci U S A.

[85] Peters CJ, Yu H, Tien J, Jan YN, Li M, Jan LY (2015). Four basic residues critical for the ion selectivity and pore blocker sensitivity of TMEM16A calcium-activated chloride channels. Proc Natl Acad Sci U S A.

[86] Pifferi S, Dibattista M, Menini A (2009). TMEM16B induces chloride currents activated by calcium in mammalian cells. Pflugers Arch.

[87] Rasche S, Toetter B, Adler J, Tschapek A, Doerner JF, Kurtenbach S, Hatt H, Meyer H, Warscheid B, Neuhaus EM (2010). Tmem16b is specifically expressed in the cilia of olfactory sensory neurons. Chem Senses.

[88] Romanenko VG, Catalan MA, Brown DA, Putzier I, Hartzell HC, Marmorstein AD, Gonzalez-Begne M, Rock JR, Harfe BD, Melvin JE (2010). Tmem16A encodes the Ca2+-activated Cl- channel in mouse submandibular salivary gland acinar cells. J Biol Chem.

[89] Ruiz C, Martins JR, Rudin F, Schneider S, Dietsche T, Fischer CA, Tornillo L, Terracciano LM, Schreiber R, Bubendorf L, Kunzelmann K (2012). Enhanced expression of ANO1 in head and neck squamous cell carcinoma causes cell migration and correlates with poor prognosis. PLoS One.

[90] Sah SP, McCluggage WG (2013). DOG1 immunoreactivity in uterine leiomyosarcomas. J Clin Pathol.

[91] Santi CM, Ferreira G, Yang B, Gazula VR, Butler A, Wei A, Kaczmarek LK, Salkoff L (2006). Opposite regulation of Slick and Slack K+ channels by neuromodulators. J Neurosci.

[92] Sauter DR, Novak I, Pedersen SF, Larsen EH, Hoffmann EK (2015). ANO1 (TMEM16A) in pancreatic ductal adenocarcinoma (PDAC). Pflugers Arch.

[93] Schreiber R, Faria D, Skryabin BV, Wanitchakool P, Rock JR, Kunzelmann K (2015). Anoctamins support calcium-dependent chloride secretion by facilitating calcium signaling in adult mouse intestine. Pflugers Arch.

[94] Schroeder BC, Cheng T, Jan YN, Jan LY (2008). Expression cloning of TMEM16A as a calcium-activated chloride channel subunit. Cell.

[95] Scott RH, McGuirk SM, Dolphin AC (1988). Modulation of divalent cation-activated chloride ion currents. Brit J Pharmacol.

[96] Scudieri P, Caci E, Bruno S, Ferrera L, Schiavon M, Sondo E, Tomati V, Gianotti A, Zegarra-Moran O, Pedemonte N, Rea F, Ravazzolo R, Galietta LJ (2012). Association of TMEM16A chloride channel overexpression with airway goblet cell metaplasia. J Physiol.

[97] Shi ZZ, Shang L, Jiang YY, Hao JJ, Zhang Y, Zhang TT, Lin DC, Liu SG, Wang BS, Gong T, Zhan QM, Wang MR (2013). Consistent and differential genetic aberrations between esophageal dysplasia and squamous cell carcinoma detected by array comparative genomic hybridization. Clin Cancer Res.

[98] Stapleton SR, Scott RH, Bell BA (1994). Effects of metabolic blockers on Ca(2+)-dependent currents in cultured sensory neurones from neonatal rats. Br J Pharmacol.

[99] Stephan AB, Shum EY, Hirsh S, Cygnar KD, Reisert J, Zhao H (2009). ANO2 is the cilial calcium-activated chloride channel that may mediate olfactory amplification. Proc Natl Acad Sci U S A.

[100] Sui Y, Sun M, Wu F, Yang L, Di W, Zhang G, Zhong L, Ma Z, Zheng J, Fang X, Ma T (2014). Inhibition of TMEM16A expression suppresses growth and invasion in human colorectal cancer cells. PLoS One.

[101] Sun H, Xia Y, Paudel O, Yang XR, Sham JS (2012). Chronic hypoxia-induced upregulation of Ca2+-activated Cl- channel in pulmonary arterial myocytes: a mechanism contributing to enhanced vasoreactivity. J Physiol.

[102] Suzuki J, Fujii T, Imao T, Ishihara K, Kuba H, Nagata S (2013). Calcium-dependent phospholipid scramblase activity of TMEM16 protein family members. J Biol Chem.

[103] Suzuki J, Umeda M, Sims PJ, Nagata S (2010). Calcium-dependent phospholipid scrambling by TMEM16F. Nature.

[104] Takayama Y, Uta D, Furue H, Tominaga M (2015). Pain-enhancing mechanism through interaction between TRPV1 and anoctamin 1 in sensory neurons. Proc Natl Acad Sci U S A.

[105] Thomas-Gatewood C, Neeb ZP, Bulley S, Adebiyi A, Bannister JP, Leo MD, Jaggar JH (2011). TMEM16A channels generate Ca(2)(+)-activated Cl(-) currents in cerebral artery smooth muscle cells. Am J Physiol Heart Circ Physiol.

[106] Tien J, Peters CJ, Wong XM, Cheng T, Jan YN, Jan LY, and Yang H (2014) A comprehensive search for calcium binding sites critical for TMEM16A calcium-activated chloride channel activity. Elife e02772 10.7554/eLife.02772 PMC4112547 24980701

[107] Tominaga M, Caterina MJ, Malmberg AB, Rosen TA, Gilbert H, Skinner K, Raumann BE, Basbaum AI, Julius D (1998). The cloned capsaicin receptor integrates multiple pain-producing stimuli. Neuron.

[108] Tran TT, Tobiume K, Hirono C, Fujimoto S, Mizuta K, Kubozono K, Inoue H, Itakura M, Sugita M, Kamata N (2014). TMEM16E (GDD1) exhibits protein instability and distinct characteristics in chloride channel/pore forming ability. J Cell Physiol.

[109] Tsukita S, Yonemura S (1997). ERM (ezrin/radixin/moesin) family: from cytoskeleton to signal transduction. Curr Opin Cell Biol.

[110] Turner JR (2009). Intestinal mucosal barrier function in health and disease. Nature Rev Immun.

[111] West RB, Corless CL, Chen X, Rubin BP, Subramanian S, Montgomery K, Zhu S, Ball CA, Nielsen TO, Patel R, Goldblum JR, Brown PO, Heinrich MC, van de Rijn M (2004). The novel marker, DOG1, is expressed ubiquitously in gastrointestinal stromal tumors irrespective of KIT or PDGFRA mutation status. Am J Pathol.

[112] Wu H, Guan S, Sun M, Yu Z, Zhao L, He M, Zhao H, Yao W, Wang E, Jin F, Xiao Q, Wei M (2015). Ano1/TMEM16A overexpression is associated with good prognosis in PR-positive or HER2-negative breast cancer patients following tamoxifen treatment. PLoS One.

[113] Xiao GN, Guan YY, He H (2002). Effects of Cl- channel blockers on endothelin-1-induced proliferation of rat vascular smooth muscle cells. Life Sci.

[114] Xiao Q, Yu K, Perez-Cornejo P, Cui Y, Arreola J, Hartzell HC (2011). Voltage- and calcium-dependent gating of TMEM16A/Ano1 chloride channels are physically coupled by the first intracellular loop. Proc Natl Acad Sci U S A.

[115] Xu Z, Lefevre GM, Gavrilova O, Foster St Claire MB, Riddick G, Felsenfeld G (2014). Mapping of long-range INS promoter interactions reveals a role for calcium-activated chloride channel ANO1 in insulin secretion. Proc Natl Acad Sci U S A.

[116] Yang YD, Cho H, Koo JY, Tak MH, Cho Y, Shim WS, Park SP, Lee J, Lee B, Kim BM, Raouf R, Shin YK, Oh U (2008). TMEM16A confers receptor-activated calcium-dependent chloride conductance. Nature.

[117] Yang H, Kim A, David T, Palmer D, Jin T, Tien J, Huang F, Cheng T, Coughlin SR, Jan YN, Jan LY (2012). TMEM16F forms a Ca2+-activated cation channel required for lipid scrambling in platelets during blood coagulation. Cell.

[118] Yu K, Duran C, Qu Z, Cui YY, Hartzell HC (2012). Explaining calcium-dependent gating of anoctamin-1 chloride channels requires a revised topology. Circ Res.

[119] Yu K, Whitlock JM, Lee K, Ortlund EA, Cui YY, Hartzell HC (2015). Identification of a lipid scrambling domain in ANO6/TMEM16F. Elife.

[120] Zygmunt AC (1994). Intracellular calcium activates a chloride current in canine ventricular myocytes. Am J Physiol.

